# Supplementary material for: Integrated information structure collapses with anesthetic loss of conscious arousal in Drosophila melanogaster
Source: PLoS Comput Biol. 2021 Feb 26;17(2):e1008722. doi: 10.1371/journal.pcbi.1008722 (PMC7946294; doi:10.1371/journal.pcbi.1008722)
Supplement: S2 Text — (PDF) [file pcbi.1008722.s002.pdf]

S2 Text. Effect of anesthesia is consistent using different timesteps

In the main text, we computed system-level integrated information and integrated information structures (IIS) from TPMs built at the timescale τ = 4 ms. We chose this timescale based on the known physiology of synaptic interactions between neurons. Specifically, if τ is too small, it will not capture causal interactions that maximise integrated information [1–3]. Thus we chose 4 ms as our timescale. A comprehensive search across τ values is infeasible due to the computational cost of system-level integrated information.

To exclude the possibility that our results vary wildly depending on the specific τ value selected, we selected a random sample of 200 channel sets (out of the total 1365 channel sets), and recomputed system-level integrated information and the IIS at two other τ values, 2 ms and 6 ms. As can be seen in Figs S2 and S3, the effect of anesthesia remains consistent with what we report in the main text (Figs 4 and 5).

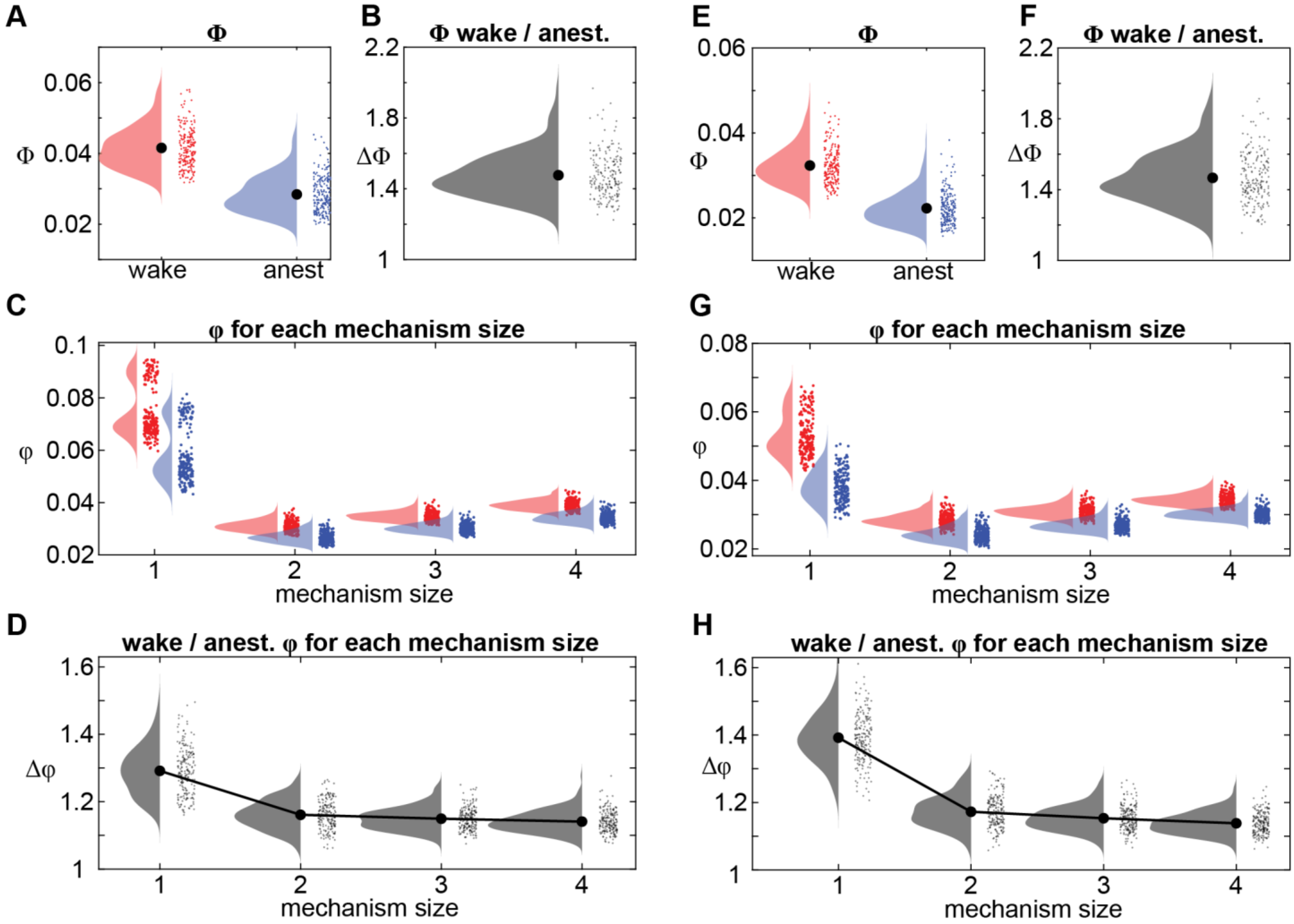

Fig S2. Effect of anesthesia on system-level integrated information (Φ) and the IIS is consistent for similar τ values. Format is the same as Fig 4 in the main article. (**A**-**D**) Results with τ = 2 ms. (**E**-**H**) Results with τ = 6 ms.

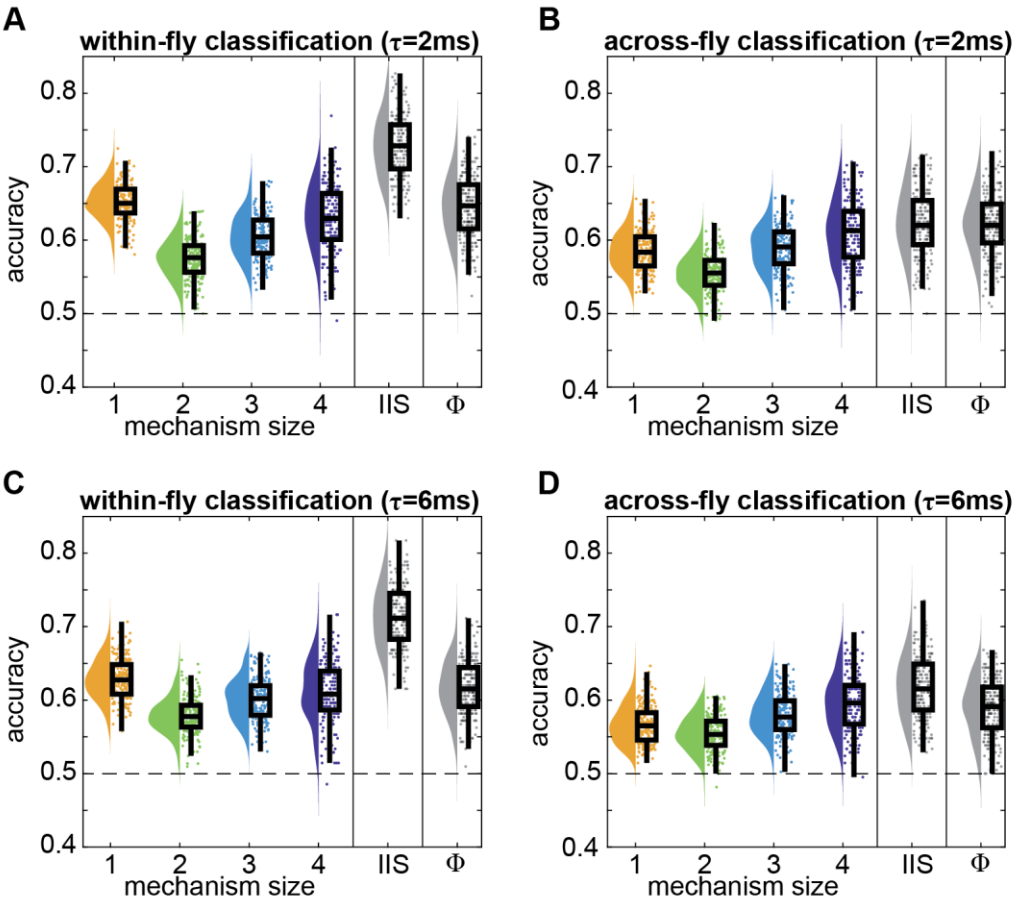

Fig S3. Classification accuracy of wake and anesthesia is consistent for similar τ values. Same format as Fig 5A and 5B in the main text. (**A**-**B**) Results with τ = 2 ms, (**C**-**D**) Results with τ = 6 ms.

References

1. Hoel EP, Albantakis L, Tononi G. Quantifying causal emergence shows that macro can beat micro. Proc Natl Acad Sci. 2013;110: 19790–19795. doi:10.1073/pnas.1314922110

2. Hoel EP, Albantakis L, Marshall W, Tononi G. Can the macro beat the micro? Integrated information across spatiotemporal scales. Neurosci Conscious. 2016;2016: niw012. doi:10.1093/nc/niw012

3. Marshall W, Albantakis L, Tononi G. Black-boxing and cause-effect power. PLoS Comput Biol. 2018;14: e1006114. doi:10.1371/journal.pcbi.1006114
