# Supplementary material for: Integrated information structure collapses with anesthetic loss of conscious arousal in Drosophila melanogaster
Source: PLoS Comput Biol. 2021 Feb 26;17(2):e1008722. doi: 10.1371/journal.pcbi.1008722 (PMC7946294; doi:10.1371/journal.pcbi.1008722)
Supplement: S7 Text — (PDF) [file pcbi.1008722.s007.pdf]

S7 Text. Recurrent connectivity is required for greater system-level integrated information

In the main text, we infer that recurrent connections throughout the fly brain is reduced by general anesthesia based on our observation that integrated information is reduced during anesthesia. A potential concern is that integrated information may be high in a nonlinear system even in the absence of recurrent connections. Here we provide a simulation to demonstrate that recurrent connectivity is required for greater system-level integrated information.

Here, we compare 2-channel integrated information among 10 simulation runs of 3 auto-regressive models with a nonlinear component: 1) a model with 2 channels that are not physically connected, 2) a model with 2 channels where one channel sends output to the other unidirectionally through a physical connection, and 3) a bidirectionally connected model (the model specifications are given below). Given these models, we would expect system-level integrated information to be greater than zero for model 3 and zero for models 1 and 2, as system-level integrated information requires bidirectional connectivity as explained extensively in [1].

The general form of these models is specified as:

- $X_{t+1} = -0.1X_t + AY_t + e_X$
- $Y_{t+1} = -0.1Y_t + BX_t + e_Y$
- Innovations covariance: diagonal 0.5, off-diagonals 0

(1) In the completely disconnected model:

- $A = 0$
- $B = 0$

(2) In the unidirectionally connected model:

- $A = 0$
- $B = 0.9$ if $X_t >$ threshold; 0 otherwise
    - (i.e., X only influences Y if X is above a certain threshold)
- threshold = 0.9

(3) In the bidirectionally connected model:

- $A = 0.9$ if $Y_t >$ threshold; 0 otherwise
- $B = 0.9$ if $X_t >$ threshold; 0 otherwise
- threshold = 0.9

We compute system-level integrated information on the simulated time series in the same way as in the main text: we 1) binarise the simulated time series based on the median, 2) obtain a TPM, then 3) use PyPhi to compute integrated information, which involves several steps as described in the main text (Fig 1 and [2]).

We find that system-level integrated information is, as expected, much greater for the bidirectionally connected model than the other two models (which are much closer to 0; Fig S8). As integrated information is always above or equal to 0, it is positively biased. While here we included a simple nonlinearity in our model (thresholds), further work should be conducted to assess the behaviour of integrated information also in partially observed systems and non-markovian systems approximated through a Markovian assumption, where spurious high-order correlations might affect the measure.

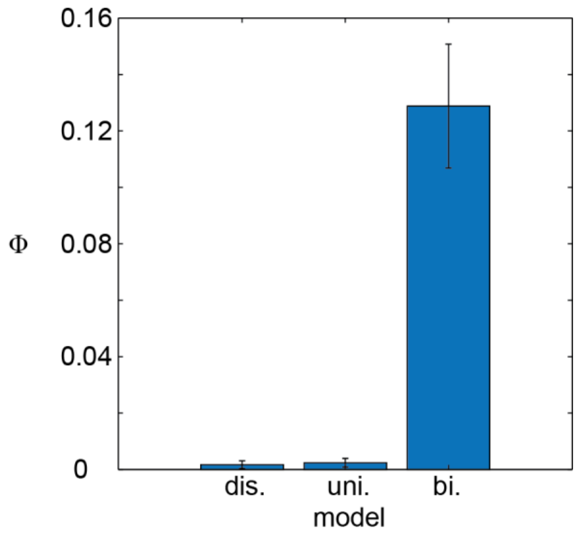

Fig S8. System-level integrated information for three simple nonlinear autoregressive models. System-level integrated information is close to 0 when the system is disconnected or unidirectionally connected. Meanwhile, system-level integrated information is much greater than 0 for the bidirectionally connected system. Shown are mean and standard deviation across 10 simulation runs of each model. For each run, a TPM was built such that each row of the TPM was obtained from observing 200 state transitions. We used these TPMs to compute system-level integrated information in the same way as we describe in the main text.

References

1. Oizumi M, Albantakis L, Tononi G. From the phenomenology to the mechanisms of consciousness: integrated information theory 3.0. PLoS Comput Biol. 2014;10: e1003588. doi:10.1371/journal.pcbi.1003588

2. Mayner WGP, Marshall W, Albantakis L, Findlay G, Marchman R, Tononi G. PyPhi: a toolbox for integrated information theory. PLOS Comput Biol. 2018;14: e1006343. doi:10.1371/journal.pcbi.1006343
