# Supplementary material for: Integrated information structure collapses with anesthetic loss of conscious arousal in Drosophila melanogaster
Source: PLoS Comput Biol. 2021 Feb 26;17(2):e1008722. doi: 10.1371/journal.pcbi.1008722 (PMC7946294; doi:10.1371/journal.pcbi.1008722)
Supplement: Attachment — Submitted filename: Responses to Editor and Reviewers.pdf [file pcbi.1008722.s009.pdf]

Dear Prof Daniele Marinazzo,

We would like to thank you for the fair evaluation of our manuscript and for being transparent about a possible course of your future action.

We carefully considered all of the constructive comments from the three reviewers, addressing all points in a point-to-point manner in the attached reply and resolving all "technical and statistical" issues, which you raised as one of the two big concerns in your letter.

Your other concern was "novel insight into the biological system". In the revised manuscript, we explicitly highlighted the novel insight into the biological system as the "integrated nature of the fly brain", which can be contrasted with a general view that the fly brain, and especially its sensory systems, is "largely feedforward". Specifically, as we have now made clearer in the manuscript, our finding suggests that integration occurs across the entire brain, including the more peripheral, sensory areas. This goes against the view that sensory areas (such as the optic lobes in the periphery of the brain) are merely providing feedforward sensory inputs to executive areas (in the central brain; Poggio and Reichardt 1976 Quarterly Reviews of Biophysics; Farris 2005 Arthropod Structure and Development), as such feedforward architecture cannot result in our finding. We have explicitly stated this by modifying the introduction and results.

We emphasized this novel biological insight in our response to Reviewer 2, by showing that integrated information of a simulated unidirectionally connected system is close to zero as in a completely unconnected system, whereas integrated information of a bidirectionally connected system is much greater than zero (Fig R2-2).

With these major revisions, we believe that our manuscript has substantially improved and now addresses all of your major concerns. Please also see attached our responses which addressed all other points raised by the three reviewers. We believe our revised manuscript is now strong and clear enough to convince all reviewers and is suitable for publication in PLoS Computational Biology.

Regards

Angus Leung & Naotsugu Tsuchiya

Reviewer #1:

We would like to thank you for the constructive comments. Below, we provide your original comments in italics and our point-by-point responses in normal font. Throughout, line numbers refer to line numbers in the revised submission.

> *Review: Integrated information structure collapses with anesthetic loss of conscious arousal in Drosophila melanogaster*
>
> *Summary:*
>
> *The authors apply Integrated Information Theory (IIT) to local field potentials (LFPs) recorded from fruit flies during wakefulness and general anesthesia. The primary result is a decrease in integrated information and the collapse of information structures during anesthesia compared to wakefulness.*
>
> *Applications of IIT to neuroimaging data are rare, and those that exist are mostly based on earlier versions of the theory (e.g., "IIT2.0"). The current work is novel in that it applies the newest version of the theory ("IIT3.0") to neuroimaging data, and it represents an important contribution to the field.*
>
> *One reason that there have been few previous applications of IIT3.0 is because it is not possible to perform the computations in large networks. For this work, the authors propose a heuristic analysis that balances theoretical motivations with practical considerations. Moreover, the authors explicitly acknowledge the limitations and caveats of their analysis. I find the work to be technically sound.*
>
> *For these reasons, I support its publication in PLoS Computational Biology. Below I list a few issues that could benefit from some clarification in a minor revision.*

We thank you for taking the time to go through our manuscript. We find your summary and context of the manuscript to be in agreement with our views. Our responses to the issues you raised are as follows:

> *Abstract:*
> *Line 29: as you point out in the discussion, your TPM is calculated by observation and not perturbation. You should not describe such interactions as causal (also other places in the Results/Methods).*

You are correct in that we calculate the TPM from observation. We modified the wording of the abstract to clarify that we are dealing with statistical estimates of causality, and not physical causality. We also modified the results section to clarify that we build the TPM from observation and not by explicitly perturbing the fly brain. As we think that using "causal" helps with the explanation of the workings of IIT, we decided to keep the term during our description of IIT, and added clarification that we are dealing with causality in the "statistical sense" and not the perturbational sense (we also added a section to the Discussion to

address the issue of observation versus perturbation, Line 678 - “Differences between perturbation and observation in building the TPM”):

- Line 31 - MODIFY:
    - “We found that **integrated interactions** among populations of neurons during wakefulness collapsed to isolated clusters of interactions during anesthesia”
- Line 184 - ADD:
    - “...”causally” (in a statistical sense) ... We refer to causality as statistically inferred from conditional probability distributions (Oizumi 2016 PNAS), which is not necessarily the same as physical causality (Pearl 2009); we return to the issue of estimating the TPM from observed versus perturbed time series in the Discussion)”

Results:
Your integrated information structure (IIS) seems very similar to the cause-effect structure (CES) of IIT3.0. When you introduce the idea, can you elaborate on how the IIS is different from the CES (is it because of the various practical approximation required?).

The IIS is indeed similar to the CES. We had reservations regarding using terms like “CES” or “constellation” exactly for the reason you suggest. Specifically, we apply necessary approximations which move us a little away from the original, ideal constructs of IIT. As we attempted to visualise in Fig 1, the IIS is a subset of all the constructs defined in IIT3.0. To help clarify this, we made the following modification:

- Line 268 - ADD:
    - “The IIS is an approximation of the full cause-effect structure proposed by IIT (Marshall 2018 PLOS Comp Bio). While the cause-effect structure requires causal intervention for building the TPM, here we only observe interactions as they naturally occur over time. Further, the full cause-effect structure holds details beyond just integrated information values, specifically the purviews of each mechanism and their associated probability distributions, whereas for simplicity the IIS only considers the integrated information values themselves.”

Lines 168-171: In IIT, this should just be the cause-effect structure. For the MICS (the M stands for maximally, not minimally), you first need to search for the set of elements that maximizes Phi.

Thanks for pointing out this mistake. We have updated the text to refer to the cause-effect structure (CES) instead of the MICS (this should also help clarify the difference between CES and IIS) when outside the context of finding the complex. An example of this change is on Line 238. This also removes the incorrect use of “minimally” that you referred to. With the change from MICS to CES, we have modified this as follows:

- Line 238 - MODIFY:

    - “...referred to in IIT as a **cause-effect structure (CES)**.”
- Line 164 - MODIFY:
    - “...terminology of “**Cause-Effect repertoire**”, “concept”, and “**Cause-Effect Structure**” (**CES**)”
- Line 550 - MODIFY:
    - “...**cause-effect structure (CES)** proposed by IIT as corresponding to the structure of consciousness…”

Methods:
I only found the value of tau in the figure caption. It would be good to include in the methods text.

Thanks for the suggestion. We added the information about tau to both the main text and methods:

- Line 193 - ADD:
    - “; we use \tau = 4 ms; we repeated analyses also at τ = 2 ms and 6 ms, see Text S2”
- Line 805 - ADD:
    - “We use \tau = 4ms...”

Also, how was the value of tau = 4ms selected?

We selected 4 ms for two reasons. First, based on the known physiology of synaptic interactions between neurons, tau which is too small (Koch 1998 Biophysics of Computation: Information Processing in Single Neurons) will not capture causal interactions that maximize integrated information (Hoel 2013 PNAS, 2016 NoC, Marshall 2018 PLoS Comp). Second, given the limited amount of our time series data, larger tau values reduce the number of transitions that we can use to compute the TPM. We chose 4 ms, which balances these two considerations and is within the range of biologically plausible timescales for synaptic interactions in the fly brains. We have added this to the methods:

- Line 805 - ADD:
    - “We use τ = 4 ms as τ which is too small will not capture causal interactions which maximise integrated information, based on known physiology of synaptic interactions (Koch 1998 Biophysics of Computation), and larger τ reduces the number of transitions that we can use to compute the TPM (but see Text S2 for repeated analyses also at τ = 2 ms and 6 ms).”

It would be good to know that similar values of tau (e.g, 2ms or 6ms) give similar results.

We have added Supplementary Text S2, where we show essentially the same results we report in the main text, but with tau values of 2 ms and 6 ms. As has been claimed by the original IIT (Tononi 2004, 2008, Oizumi 2014), integrated information should be computed at the timescale where system-level integrated information is maximal. We are currently

working on this issue using a combination of analytical models, computer simulation, and validation through real data. However, we believe this is beyond the topics that we can cover in this paper. Thus, we briefly mention the issue of timescale (and provide our supplementary result) in the Discussion ("Role of system-level integrated information" - Line 664).

> *Were separate TPMs for each 2.25s epoch? Or were the epochs somehow combined to create a single TPM? This was not clear for me.*

Yes, we built separate TPMs for each 2.25s epoch, computed system-level integrated information and the IIS, and then averaged across epochs. For the across-fly classification analysis, we kept the trials as a source of variance. To make this clearer in the text, we made the following modifications:

- Line 822 - ADD:
    - "We computed the state-by-channel TPMs for every possible, 4-channel subset out of the 15 channels (15choose4 = 1365 channel sets), repeating this procedure for each fly and epoch (obtaining one TPM per fly and 2.25s epoch)."
- Line 835 - ADD:
    - "For the comparison of integrated information values between wakefulness and anesthesia, we further averaged these values across the 8 epochs."

> *Discussion:*
> *Regarding the claim that the IIS is more reliable than Phi for assessing level of consciousness. A potential pitfall here is that there is nothing to stop non-integrated systems from having many large small phi values. For example, if you took 4 channels from one fly, and four channels from another fly, then you could have lots of mechanisms with non-zero small phi, but the whole system would not be integrated (and the two flies presumably not jointly conscious). That there were in fact two systems is something you would only see with Phi. In your situation, it works out okay because all the systems you analyze are integrated to some degree, but it would be good to address this.*

We agree. To highlight the continued relevance of system-level integrated information, in light of better classification performance using the IIS, we added a section to the discussion (Line 650 - "Role of system-level integrated information").

Reviewer #2:

We would like to thank you for your constructive comments. Below, we provide your original comments in italics (with bolding and numbering for clarification and our point-by-point responses in normal font. Throughout, line numbers refer to line numbers in the revised submission unless otherwise specified.

> *This paper claims to show that system-level integrated information is reduced in the fly brain during anesthesia, and that structures of integrated information collapse in the anesthetized state. These results are interpreted as confirming the integrated information theory of consciousness.*

We thank you for taking the time to go through our manuscript. We find your summary of our results to be accurate. However, we do not make such a strong interpretation as taking our results to be confirming IIT. Instead, we interpret our results as "consistent with IIT's predictions", with caveats from applying the theory to real data (which we acknowledged in the old Discussion, Line 435 of the original manuscript, and have now expanded on in the new Discussion section "Differences between perturbation and observation in building the TPM", Line 678). We went through the manuscript to try and identify passages which may have suggested a stronger interpretation, but were unable to identify any obvious passages. If we have erroneously made such a statement, please let us know the line number(s) and we will modify it.

Our responses to the specific issues you raised are as follows:

> *As it stands, I am not convinced by this analysis, for several reasons.*
>
> *The first reason is conceptual, and centers on the difficulty of inferring the presence, absence, or character of consciousness in the fly brain. To begin, it is far from clear* ***whether flies are conscious at all*** *during waking states. Moreover, if flies are conscious during waking states, then it still is impossible to infer* ***what the phenomenology of that consciousness is like****. This is a major issue for the reported analyses, because the integrated information theory of consciousness - on which the reported analyses are based -* ***begins with the phenomenology of human consciousness.*** *In particular, the theory begins with the observation that human consciousness forms an integrated whole, i.e. that* ***percepts across all of our sensory modalities are simultaneously available to us.*** *This phenomenological observation serves as the basis for the mathematical structure of the theory, and those are the mathematical structures used here to analyze the fly brain. Problematically, we cannot know if this integrated phenomenology is present in flies, even if we assume that flies have some form of subjective experience. And* ***there is reason to doubt that flies experience an integrated perceptual whole, because it is not even clear that all vertebrates experience integrated perception****. For example, extensive experiments on the visual systems in frogs suggest that frogs' visual perception is not integrated as ours is (see Chapter 1 of Milner and Goodale's The Visual Brain in Action). And, while there is some evidence*

of multi-sensory integration in the mushroom bodies of insect brains (see, e.g., Farris 2002, "Evolution of insect mushroom bodies: old clues, new insights") and in the ventrolateral lobes of a few insects (see, e.g., Anton et al 2011, "Brief predator sound exposure elicits behavioral and neuronal long-term sensitization in the olfactory system of an insect"), in general it's thought that ***sensory processing in insect brains is not as integrated as it is in vertebrate brains*** *(see Chittka and Niven 2009, "Are* Bigger Brains Better?"). *In summary, even if the integrated information theory of consciousness is true, it is still unclear* ***1) whether we should expect a priori that flies are conscious during waking states****, that* ***2) their experience is integrated during waking states****, that* ***3) they are unconscious under anesthesia****, and that their* ***4) unconsciousness under anesthesia is due to a collapse of information integration****.*

We have numbered the key issues which you have brought up here, and bolded the key concepts leading to them.

For points 1) and 3), as to "Whether we should expect a priori that flies are conscious during waking states" and "(whether) they are unconscious under anesthesia", we believe that accumulating and converging evidence suggests that waking states involve some aspects of consciousness in flies, which are lost under general anesthesia at the dose that we applied. However, we admit that these philosophical questions are not something that our data alone can answer.

While we cannot be 100% sure of the presence of consciousness in flies, and non-human animals in general (and actually, in principle, also in humans other than ourselves), the fundamental approach of answering this question is to search for behavioral or physiological similarities between humans and the non-human animals in question. Given sufficiently strong behavioral and physiological similarities, which are supported by genetic and phylogenetic continuity in biology, we may, at some point, consider that the weight of evidence favors attributing consciousness to that particular animal or species (Boly et al., 2013 Frontiers in Psychology; Mashour and Alkire 2013 PNAS).

From this viewpoint, we would like to point out that there is increasing evidence to support many similarities identified between insects, including flies, and mammals, including humans. With respect to conscious arousal, multiple similarities have been found in terms of different states of arousal and how sleep and anesthesia are regulated in insects (primarily flies) and mammals (Cirelli and Bushey 2008 Annals of the New York Academy of Sciences; Kirszenblat and van Swinderen 2015 Trends in Neurosciences; Barron and Klein 2016 PNAS; Cohen 2018 eNeuro). With respect to psychological processes that are closely related to consciousness in humans, insects and mammals have been shown to exhibit multiple homologous functions, behaviours, and neural correlates, such as illusory contour perception (bees; Horridge et al 1992 Philos Trans), feature binding (flies; Grabowska et al. PNAS in press), attention (flies; van Swinderen, 2011 International Review of Neurobiology; Kirszenblat and van Swinderen 2015 Trends in Neurosciences; bees; Nityananda and Chittka 2015; Chittka and Wilson 2019 American Scientist; dragonflies; Wiederman 2013 Current Biology), working memory (flies; Greenspan and van Swinderen, 2004),

metacognition (bees; Perry and Barron 2013 PNAS), false memory (bees; Hunt and Chittka 2015 Current Biology) and long-term future planning (bees; Gallo and Chittka 2018 Frontiers in Psychology). While these examples of converging evidence is interesting and encouraging to us, our manuscript specifically targets levels of consciousness, and understanding the potential contents of fly consciousness is outside its scope. Taken altogether, while there is much to clarify, we believe that a simpler notion of varying levels of some kind of conscious arousal (regardless of its contents) in flies is less controversial.

We have decided to add these considerations into the Introduction (Lines 68-73, 103-112, 116) and Discussion (Line 700 - "Applying IIT to loss of consciousness in flies"). Given that fly consciousness is not firmly established, we also decided to refer to "level of arousal" rather than "conscious level", when directly discussing the fly (we still refer to consciousness in general when discussing the underlying motivation and ideas of IIT, etc.). For example:

- Lines 122 - MODIFY:
    - "...utility of information structure as a measure for level of **arousal**"

For point 2), whether "their experience is integrated during waking states" is not a critical question for IIT and our paper (as IIT does explicitly acknowledge non-integrated conscious phenomenology in humans and other conscious animals via the concept of "complex"; note that we did not explain this idea of "complex" in the original manuscript).

IIT explains this idea of non-integrated phenomenology in wakeful normal healthy subjects through the concept of a "complex", the set of system parts which maximise system-level integrated information (we now describe this in an addition to the Discussion, "Role of system-level integrated information" - Line 657). If some sensory processing in the brain is not included in the "complex", it is not a part of conscious experience; it is unconscious neuronal processing. Rather, only the sensory modalities which are integrated within the complex would contribute to conscious experience. If, as you suggest, the complex of the fly brain is indeed less integrated than the complex of vertebrate brains, system-level integrated information should be larger in the vertebrate and smaller in the fly.

This idea of the complex has some support from experiments performed with human subjects. For example, Sasai 2016 PNAS analysed fMRI data, testing the possibility that subjects are not consciously aware of the auditory modality while they are focusing on a demanding visuo-motor integration task. Their analysis suggested that unconscious auditory processing during the task was indeed outside of the complex. The idea of the complex in IIT can potentially explain other cases of unconscious processing such as visual agnosia and blindsight (Whitwell 2014 Frontiers in Neurology; Celeghin 2015 Consciousness and Cognition), as well as the examples discussed in Milner & Goodale's book.

If we were to analyse the complex in flies, we might be able to address the issue of whether or not their experience is integrated during waking states. If, as you suggest, the complex of the fly brain is indeed less integrated than the complex of vertebrate brains, system-level integrated information should be larger in the vertebrate and smaller in the fly. However, properly identifying the complex in IIT 3.0 is computationally extremely costly. While we have

plans to apply other, recently proposed, methods (Hidaka & Oizumi 2018 PLoS One, Toker 2019 PLoS Comp, Kitazono 2018 Entropy) to analyse the complex in fly brains, these methods were developed for a previous version of IIT and do not extend to IIT 3.0. Given these considerations, and our original aim of assessing informational structures instead of just the scalar system-level integrated information, we consider analysing the complex of the fly brain to be outside the scope of this paper.

For point 4), as to whether "their unconsciousness under anesthesia is due to a collapse of information integration", in the paper we do not claim that the latter causes the former (however, IIT does make a theoretical claim that the two are "identical", or "explanatory"). Rather, the reverse in logic (approximately) is what we found. As we claimed in the subsection heading ("Integrated information structure collapses due to anesthesia" - Line 368), we found that the integrated formation structure collapsed *due to* general anesthesia (which we manipulated). For the future, it may be interesting (and possible to do with flies) to see if complex behaviors that are strongly associated with consciousness in humans are lost if we just disrupt the integrated information structure using optogenetics, without changing other possible neural activities. Overall, we do not think this should be considered a "conceptual issue" of our paper.

Regarding the specific analysis issues you raise, we provide our point-to-point replies below.

> *Aside from these conceptual issues, there are also a number of major analysis issues in this paper.*
>
> *The first problem, which the authors note in the Discussion, is that the recorded local field potentials are discretized through* ***a simple binarization at the median****. The transition probability matrices which are used to calculate integrated information are then estimated based on this discretization. While such binarization has been employed elsewhere, for e.g. in computing the Lempel-Ziv complexity of brain signals, it is unclear* ***whether such a binarization*** *would yield accurate results in the estimation of integrated information, particularly for a continuous, nonlinear signal like a local field potential. Presumably, the structure of state transitions in the fly brain is far more complex than can be captured through* ***a simple binarization****.*

While we can potentially operationalise the states of the brain signals in many different ways, binarization at the median is the simplest discretisation process, and as you point out, has been used for other measures (we have also successfully used this process to investigate information structures in fly brain in Munoz 2020 Physical Review Research). Further, binarization specifically at the median normalizes entropy across all epochs. This is important as it controls for potential changes in entropy levels between wakefulness and anesthesia (which relates to another concern that you raise later).

To quantify the effects of a simple binarization at the median on the estimation of integrated information, we have tried binarization using different thresholds (30th up to 70th percentiles, in steps of 5), and comparing system-level integrated information for sets of 2 channels at a time (not 4 channels at a time, due to computational cost). We found the effect of anesthesia

on 2-channel system-level integrated information to be consistent regardless of threshold (Fig R2-1). We have decided to show this as a supplementary result (Text S1, referred to in Line 131).

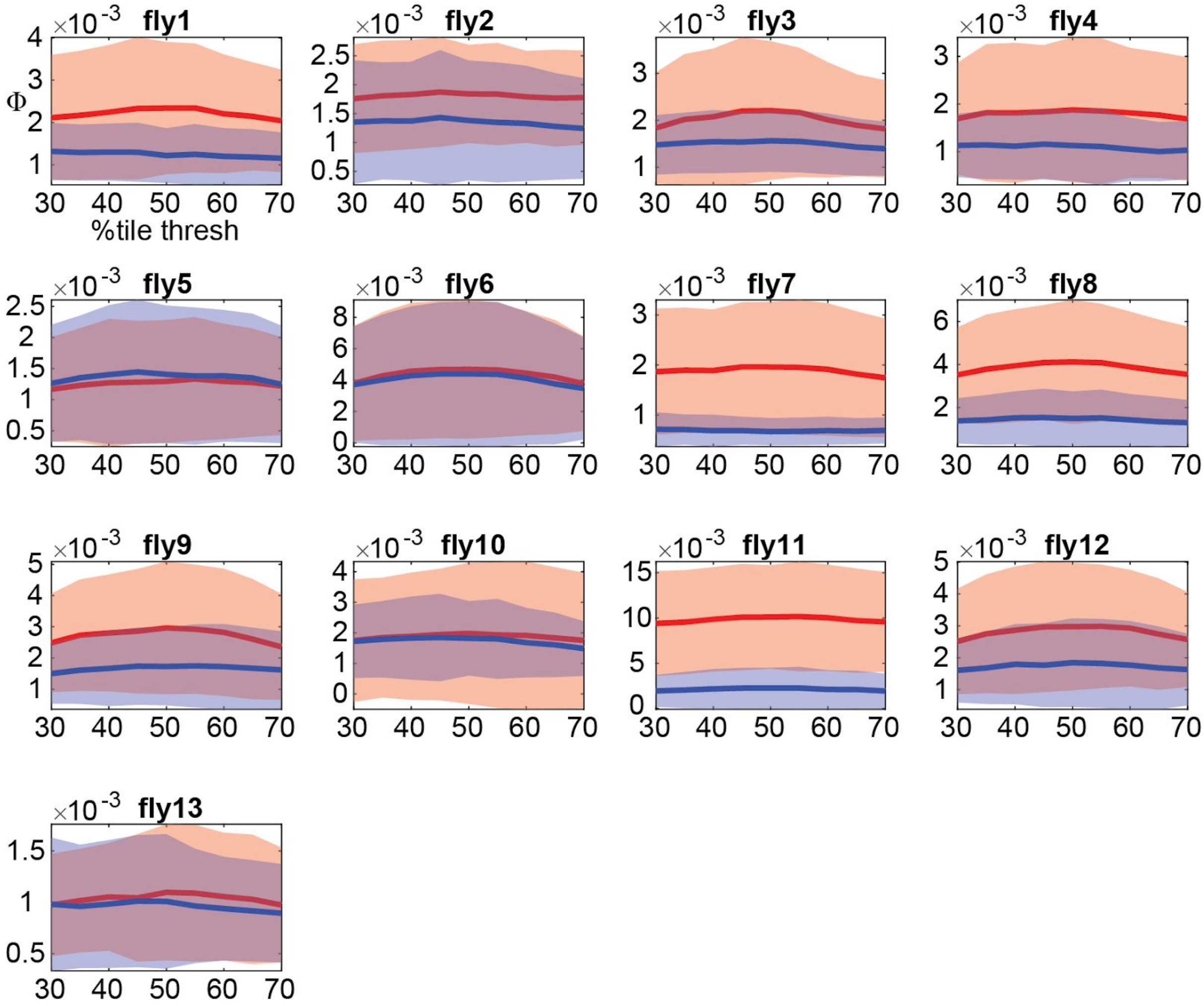

Fig R2-1. Effect of anesthesia on system-level integrated information is consistent across different binarization thresholds. We compute system-level integrated information (for sets of 2 channels at a time) after binarising voltages of each channel at a given threshold (30th up to 70th percentiles; voltages become ‘1’ if above the threshold, and ‘0’ otherwise). Plotted is mean and standard deviation (across 105 channel sets per fly), for wake (red) and anesthesia (blue).

> *Moreover, it is unclear how the authors estimate the transition probability matrix of the “statistically disconnected” fly brain. As the authors note, the calculation of integrated information requires comparing the transition probability matrix of a full system to the transition probability matrix of that same system, once that system has been severed at its weakest informational link. A* ***“statistical” estimate of the behavior*** *of a system following a network cut is possible only for linear, Gaussian variables. But, because the dynamics of the fly brain are presumably nonlinear, it is, by definition,* ***impossible to estimate the transition probability matrix of the disconnected*** *fly brain without* ***actually physically severing neural connections*** *in the fly brain - and doing so for all possible partitions or bipartitions of the brain.*

Here we think there are two misunderstandings (please correct and clarify if we have misinterpreted your concerns here). The first is regarding the purpose of the TPM, and the second is regarding the nature of the disconnected TPM.

While our description of the TPM may have suggested that its goal is to estimate the "behaviour" of a system, this is not in fact its purpose in IIT. Rather, the TPMs in IIT are used for estimating how much some state of the system (or its mechanisms) constrains (i.e. informs about) the possible past and future states of the system. "Constraining" past and future states is a bit abstract, but it is in the vein of making a statement such as "given that this neuron fires at time t, these postsynaptic neurons will likely fire at time t+\tau". Conversely, a lack of "constraining" is in the vein of "given that this neuron fires at time t, we don't know whether these other neurons will fire at time t+\tau". This "constraining" (i.e. reduction of possible outcomes) is equivalent to the notion of "information" used in standard information theory (Shannon 1948). The purpose of the TPM, therefore, is not to describe "actual" behaviours of a system, but to estimate how its behavioural repertoire is constrained by its states (i.e. to estimate the information each state has about the system's behavioural repertoire).

Following this, the goal of a "disconnection" is to quantify the degree to which "interactions" among parts of the system (or its mechanisms) cannot be factored out into statistical independent subsets (Tegmark 2016 PLoS Comp). The central idea in IIT (and other information geometry techniques; Oizumi 2016 PNAS) is to measure the degree of interactions by a statistical "disconnection", which can be applied to linear and nonlinear systems, without any requirement of Gaussianity. The statistical disconnection replaces interactions among parts with random noise, such that states of some part of the system or a mechanism no longer constrains the past or future states of some other part.

We realise that the term "disconnection" may be misleading, in that it may imply requiring physical disconnections. This is why, in the main text, we introduce "disconnection" with the term "statistically noised" (Line 207). To help further clarify how a disconnection is made, we added Supplementary Material Text S3 (referred to in Line 207), where we explicitly walk through an example of what we mean by "disconnection" (i.e. "statistically noised").

As we think the misunderstanding regarding the role of the TPM arises from how we originally introduced the TPM (specifically, immediately after introducing the TPM we say "The TPM characterizes how the whole system evolves over time"), we have made the following modifications to clarify the purpose of the TPM:

- Line 188 - ADD:
    - "Importantly, we use the TPM to measure the information generated by the system when it is in a particular state"
- Line 200 - ADD:
    - Add - "Such a probability distribution specifies the information generated by a mechanism over a given purview."

> *Moreover, even if the authors' method for statistically estimating the transition probability matrix of the disconnected fly brain were valid, they do not perform this for*

all possible cuts of the system, as they note on lines 183-188. What guarantee do we have then that their estimate is close to the ground-truth?

For all of our computations, we do in fact consider all possible cuts. However, we realise that our original sentences may have been misleading. To clarify this, we made the following modification:

- Line 259-276 - MODIFY:
  - “One difficulty with Φ is the high computational cost due to the combinatorial explosion of all possible system cuts. To enable us to search through all possible cuts, we restricted analysis to 4 channels at a time, using every combination of 4 channels as a “system”. This provides a good balance between spatial coverage for each set of channels and computation time” …. “We also considered a computationally cheaper alternative to Φ. Specifically, we assessed a set of φ values, which we term Integrated Information Structure (IIS; Fig 1I), as an alternative measure for discriminating the level of consciousness. A set of mechanism-level φ values are faster to compute, as they are already obtained as part of the computation of Φ.”

Considering the number of heuristics used in this paper’s methods - namely, ***1) binarization****, **2)** statistical estimation of the* ***disconnected transition probability matrix****, and* ***3) searching through only a sub-sample of partitions*** *- I would need to see much more evidence that these heuristics approximate the ground-truth in at least a simulation of a complex, nonlinear system before I am convinced that these heuristics approximate the ground-truth in empirical recordings from a complex, nonlinear system.*

To summarize our replies so far, we 1) addressed the effects of binarization in Figure R2-1, 2) clarified the meaning and procedure of the disconnected TPM above, and 3) searched through all partitions within each 4-channel system.

Having said that, we here address the additional request from the reviewer - the comparison with “ground-truth” in a simulation. We investigated a nonlinear auto-regressive model. In Fig R2-2 we compare 2-channel system-level integrated information among 10 simulation runs of 3 models: 1) a model with 2 channels that are not physically connected, 2) a model with 2 channels where one channel sends output to the other unidirectionally through a physical connection, and 3) a bidirectionally connected model (the model specifications are given below). Given these models, we would expect system-level integrated information to be greater than zero for model 3 and zero for models 1 and 2, as system-level integrated information requires bidirectional connectivity as explained extensively in Oizumi 2014 PLOS Comp Bio.

We compute system-level integrated information on the simulated time series in the same way as in the manuscript: we 1) binarise the simulated time series based on the median, 2) obtain a TPM, 3) marginalise to obtain “disconnected” (i.e. noised) TPMs, and 4) compare the probability distributions from the TPM with those from the disconnected TPMs. We find

that system-level integrated information is, as expected, much greater for the bidirectionally connected model than the other two models (which are much closer to 0). While we think this simulation is outside the scope of this paper, we are happy to consider incorporating it if you and the editor recommend it.

The full general form of the models is specified as:

- $X_{t+1} = -0.1X_t + AY_t + e_X$
- $Y_{t+1} = -0.1Y_t + BX_t + e_Y$
- Innovations covariance: diagonal 0.5, off-diagonals 0

(1) In the completely disconnected model:

- A = 0
- B = 0

(2) In the unidirectionally connected model:

- A = 0
- B = 0.9 if X_t > threshold; 0 otherwise
    - (i.e., X only influences Y if X is above a certain threshold)
- threshold = 0.9

(3) In the bidirectionally connected model:

- A = 0.9 if Y_t > threshold; 0 otherwise
- B = 0.9 if X_t > threshold; 0 otherwise
- threshold = 0.9

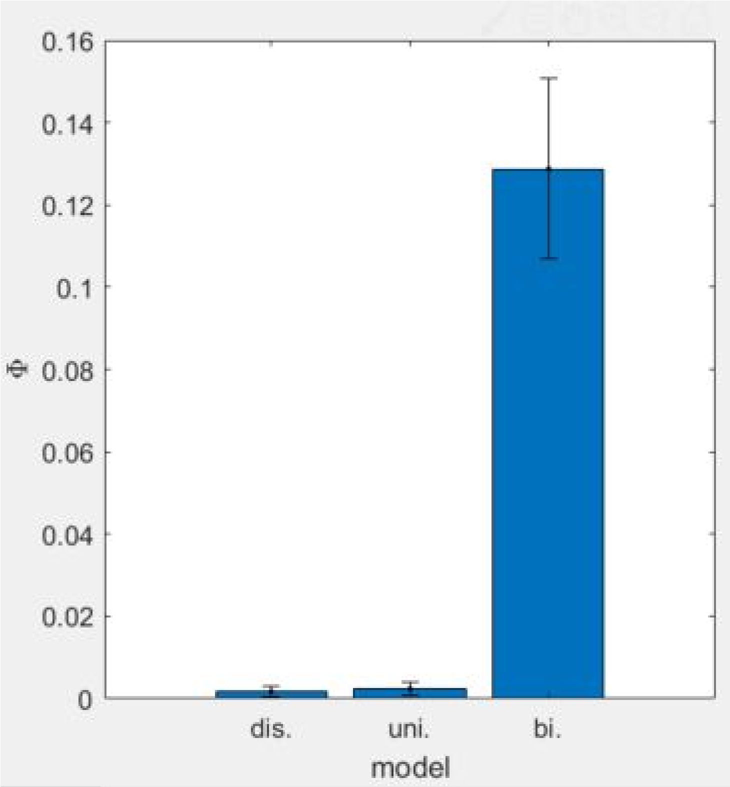

Fig R2-2. System-level integrated information for three simple nonlinear autoregressive models. System-level integrated information is close to 0 when the system is disconnected or unidirectionally connected. Meanwhile, system-level integrated information is much greater than 0 for the bidirectionally connected system. Shown are mean and standard

deviation across 10 simulation runs of each model. For each run, a TPM was built such that each row of the TPM was obtained from observing 200 state transitions. We used these TPMs to compute system-level integrated information in the same way as we describe in the main text.

> *Finally, I would need to see more evidence that the effects reported here are* ***not simply driven by changes to the entropy*** *of the flies' local field potentials under anesthesia. There is evidence that reports of changes to neural information transfer under anesthesia may in fact be driven by drops in local entropy (see Wollstadt et al 2017, "Breakdown of local information processing may underlie isoflurane anesthesia effects."). In other words, it may be that the mechanisms of integration/communication are unaffected by anesthesia, and that anesthesia instead disrupts how much information is available to be integrated/communicated. Reports of a breakdown of information transfer or communication under anesthesia may therefore be erroneous. In light of Wollstadt et al's findings,* ***I would like to see that the results reported in this paper are not driven by changes to entropy****. This may be done by, for e.g., normalizing estimates of integrated information by estimates of entropy, and showing that these normalized estimates are likewise reduced under anesthesia, or by statistically showing that variance in integrated information cannot be explained by variance in entropy. On a related note, I wonder if a disruption to local entropy may be driving the authors' finding that 1-channel mechanisms are the most strongly affected by anesthesia.*

Note that, because we used median split for binarizing voltages at each epoch, the probability of each channel being "on" and "off" was 0.5. This is true for all epochs, both wake and anesthesia, and all flies. Thus, a simple measure of the entropy (as in local entropy in Wollstadt et al 2017) of the binarized LFP is exactly the same in all flies, for both conditions. Further, because entropy is equal in all epochs, our finding that 1-channel mechanisms are most strongly affected by anesthesia is not driven by some disruption in entropy. We now explicitly mention equal entropy among epochs due to binarization using the median in Line 669).

> *Based on these issues, I recommend rejection or otherwise major revision of this paper.*

Again, we thank you for reading our manuscript and raising these important issues. We hope our clarifications, responses and modifications have been able to satisfactorily address the concerns you raised.

Reviewer #3:

We would like to thank you for the constructive comments. Below, we provide your original comments in *italics* and our point-by-point responses in normal font. Throughout, line numbers refer to line numbers in the revised submission unless otherwise specified.

> *## Summary*
>
> *In this paper, Leung et al apply measures inspired by a version of integrated information theory (IIT 3.0) to LFP data of awake and anesthetised flies. The main result is that system-level integrated information \Phi and mechanism-level integrated information \phi decrease under general anesthesia. Furthermore, the authors show that classifiers built on the whole set of \phi (an integrated information structure, IIS) is better at predicting whether flies were anesthetised than classifiers built on \Phi or \phi alone.*
>
> *The main contribution of this paper is the first application of a measure inspired by IIT 3.0 to neural data. I would not say that this is IIT 3.0, because the theory relies on many assumptions (Markovianity, full observability, causal perturbations) that do not hold in this analysis, and involves calculation steps that have not been taken here (the authors mention this in the Discussion). The paper is well written, and data has been made publicly available. Although the results seem interesting, I have a few methodological concerns that must be addressed before the quality and significance of the paper can be fully judged.*

We agree with you that we need to be careful in writing with respect to whether our paper should be considered as a “strict” application of IIT 3.0 to real data. We modified the text to help clarify the difference between what we did and what IIT 3.0 ideally requires as follows:

- Line 31 - MODIFY:
    - “We found that **integrated interactions** among populations of neurons during wakefulness collapsed to isolated clusters of interactions during anesthesia”
- Line 184 - ADD:
    - “...”causally” (in a statistical sense) ... We refer to causality as statistically inferred from conditional probability distributions (Oizumi 2016 PNAS), which is not necessarily the same as physical causality (Pearl 2009); we return to the issue of estimating the TPM from observed versus perturbed time series in the Discussion)”
- Line 268 - ADD:
    - “The IIS is an approximation of the full cause-effect structure proposed by IIT (Marshall 2018 PLOS Comp Bio). While the cause-effect structure requires causal intervention for building the TPM, here we only observe interactions as they naturally occur over time. Further, the full cause-effect structure holds details beyond just integrated information values, specifically the purviews of each mechanism and their associated probability distributions, whereas for simplicity the IIS only considers the integrated information values themselves.”

We have also expanded the Discussion to include more details on what a completely faithful (but infeasible at the moment) application of IIT 3.0 would require (Line 650 - “Role of system-level integrated information” and Line 678 - “Differences between perturbation and observation in building the TPM”).

Our responses to the other issues you raised are as follows:

> *## Major comments (most important first)*
>
> *1) Statistical reporting*
>
> *My main concern with the paper is the inappropriateness of the statistical reporting. Most prominently, the paper reports some incredibly small p-values (e.g. t-values of -85), which I believe are strongly misleading. When used in this kind of studies, p-values typically refer to group-level comparisons, and not to the p-value of coefficients of the model (with repeated measures, etc). The method used by the authors leads to excess degrees of freedom that are radically underestimating the p-values of the results. As an example, in the first reported LME model in L.207 the actual number of degrees of freedom is much closer to 13 (due to the correlation between \Phi values in the same fly) than to 35488.*
>
> *In general, the authors should report more comprehensive statistics, and avoid misleading p-values. This includes, but is not limited to:*
>
> *- Performing t-tests with 13 degrees of freedom of quantities of interest (e.g. \Phi, within-fly class. accuracy) averaged across epochs and channel sets.*
>
> *- Showing more descriptive statistics of \Phi, like mean \Phi and effect size of anesthesia for each fly.*
>
> *- Reporting standard deviation of random effects in the LMEs.*
>
> *- Reporting R^2 for the LMEs.*

We agree with the concern regarding small p-values and high degrees of freedom. This issue arises in the way we have reported post-hoc comparisons after first conducting omnibus tests (and confirming significant effects) using likelihood ratio tests comparing LME models. As far as we are aware, there is no clear way of performing post-hoc comparisons which take into account the nested nature of the data. As a compromise, we resorted to reporting the t-statistics associated with fitted coefficients of restricted LME models (when restricting the effect of interest to 2 levels at a time; and using the random effects structure to account for nesting). This is similar to how one can conduct a t-test using linear regression, but we utilise the random effects structure offered by LME. Note that our statistics were obtained through MATLABs implementation of LMEs - we did not calculate these independently.

While we describe this in the Methods section (Line 572 in the original manuscript), we understand that our first statistical result being reported in a similar fashion to a standard group-level t-test may confuse readers. To address this concern, we decided to follow your suggestions and replace these stats with more standard group-level tests. Specifically, we

now report uncorrected t-tests, comparing values after averaging across epochs and channel sets. As an indicator of effect sizes for these tests we have decided to provide the LME coefficients when fitting two groups at a time. We have updated the Statistical analyses section to reflect the new tests:

- Line 894 - MODIFY:
    - "To conduct pairwise comparisons (e.g. to compare 1-channel to 2-channel integrated information), we limited the effect of interest to two levels at a time and report the associated regression coefficient. As *p*-values associated with these regression coefficients were very small and potentially do not reflect the true degrees of freedom, we report the coefficients along with a group-level *t*-tests (conducted after averaging across channel sets to obtain a single value per fly or, for across-fly classification, per epoch)."

An example of the change in the reported statistics is:

- Line 307 - instead of "$t(35488) = -85.57$, $p < .001$", we write "$\beta = -0.012$ $t(12) = -2.525$, $p = .013$, one-tailed"

After shifting to more standard group-level tests for pairwise comparisons, the majority of results remain significant. The new statistical tests have changed the results in the following 4 cases:

- Within-fly classification
    - Line 459: 1-channel mechanism average classification performance is comparable to system-level integrated information performance
    - Line 458: 1-channel mechanism average classification performance is comparable to 4-channel mechanism performance
- Across-fly classification
    - Line 500: system-level integrated information classification performance is comparable to IIS performance
    - Line 506: system-level integrated information classification performance is comparable to 4-channel mechanism performance

These changes do not affect the main findings of the manuscript - specifically that anesthesia reduces system-level integrated information and collapses the IIS across the fly brain.

As you also suggested, we have included descriptive stats at the individual fly level for system-level integrated information, in Supplementary Material Text S4 (referred to in Line 310), and model R^2 and standard deviation of random effects for LME models in Table 2 (referred to in Line 909, shown at Line 963).

2) Control for model size in IIS classification

One of the central results of the paper is that classifiers using IIS as features are better than classifiers that use \Phi or \phi. However, while from Fig 5 it visually seems to be the case (at least for within-fly classification), the paper does not provide conclusive evidence that IIS are significantly better, since it is to be expected that classifiers built on IIS

may perform better just by virtue of having more trainable parameters.While the paper does report some p-values related to the classifiers (L.312 and below), I suspect it may be another instance of the issue with the p-values I pointed out above. To have more convincing evidence that the IIS is indeed statistically preferred as a predictor, the authors should use standard model selection techniques for each fly/channel set, confirm that the IIS model is always preferred (using standard criteria like AIC or BIC), and report the results explicitly.

Our central claim that the IIS performs better than /Phi or /phi is based on the prior expectation that the IIS should be more reflective of consciousness per se than /Phi as a summary or individual /phi values which ought to reflect “parts” of the contents of consciousness.

We have added a supplementary analysis (Text S5, referred to in Line 477) where we conduct logistic regression of \phi values or \Phi with wakefulness/anesthesia for each fly (using channel sets as observations) and compare the AIC values of the models. Summarily, by fitting models using either only 1-, 2-, 3-, or 4-channel mechanisms, all mechanisms (i.e. the IIS), or system-level integrated information, we find that the IIS model has the lowest AIC for all flies (Fig R3-2).

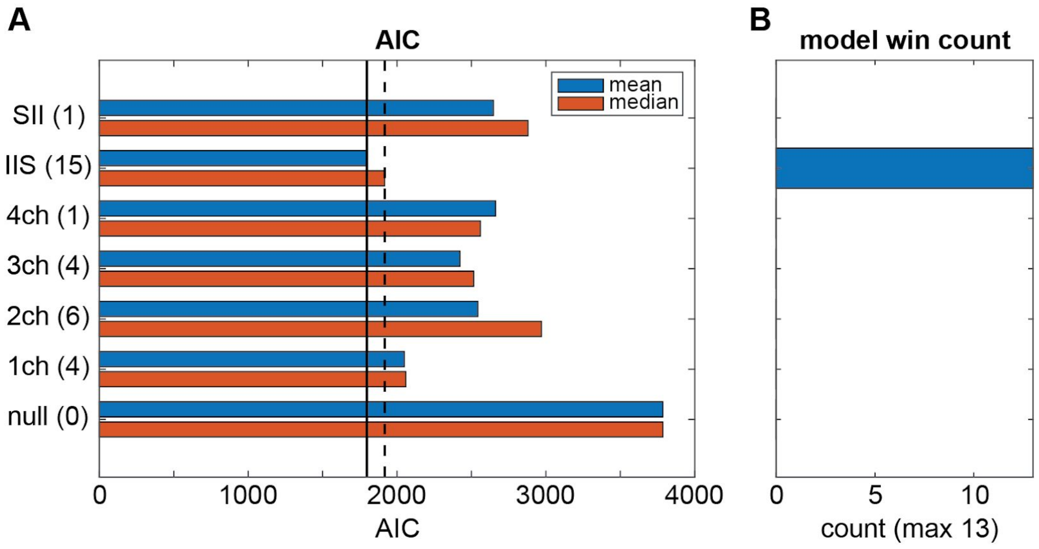

Fig R3-2. AIC values for regressing wakeful/anesthetized conditions onto different mechanism sizes, and system-level integrated information. Null models regress wakeful/anesthetized conditions onto only an intercept. (A) AIC from fitting models from 1365 channel sets per fly (observations per model = 1365 channel sets x 2 conditions). Shown are mean (blue) and median (red) of the 13 AIC values obtained from each of 13 flies. Solid and dashed lines indicate that the full IIS model performs the best (i.e. gives the smallest AIC) in terms of the mean of the median. Y-labels give the features used for fitting the model and the associated number of coefficients fitted (in parentheses), excluding the intercept. (B) The full IIS model is chosen as the best model, which gives the minimal AIC in all 13 flies, while the SII model is never chosen as the best model.

The convergence of the results between our leave-one-out cross-validation analysis (Fig 5A) and the model selection analysis with AIC is consistent with the analytical relationship between cross validation and AIC (Stone 1977 Journal of the Royal Statistical Society: Series B (Methodological), Fang 2011 Journal of Data Science).

3) Single-mechanism \phi

The high values and strong effects obtained for single-mechanism \phi are indeed, as the authors point out, very unexpected. While these do not contribute to system-level \Phi directly, they do form an important part of the IIS and therefore play a role in the paper's main results. Since they are certainly "not a well-developed theoretical construct", the authors should elaborate more on what they are, what they mean, and how they contribute to the results.

For example, the authors could make sure that this single-mechanism \phi does not contribute to the IIS classification accuracy by repeating the analyses with IIS made of only sets of two or more mechanisms.

We have now added some speculation as to how 1-channel results might be interpreted in Discussion.

- Line 636 - ADD:
  - "Specifically, integrated information for a mechanism is assessed by comparing the information it generates before and after imposing some disconnection among its parts. 1-channel mechanisms however cannot be split and compared in this manner. While IIT 3.0 specifically considers disconnections between a mechanism and its purview, and so some disconnection can always be imposed for any mechanism-purview combination, disconnections must still separate the mechanism into independent parts (each affecting their own independent purviews) (Albantakis 2019 Entropy), and thus the problem remains. In Fig 1G, we illustrate that the purviews of mechanisms A and B were simply themselves. In this example, imposing a disconnection on these self-connections seemed to result in a relatively large loss of information (compared to mechanism AB). While further investigation is necessary to understand our finding regarding 1-channel integrated information, our main results regarding the IIS are unaffected, as we verified that 1-channel mechanisms were not driving its classification performance (Text S6)."

As you suggested, we also clarified that 1-channel mechanisms do not drive our results regarding classification of wake/anesthesia using the IIS. We repeated the classification analysis using the IIS, but excluding 1-channel mechanisms. Summarily, the restricted IIS (i.e. IIS consisting only of 2-, 3-, and 4-channel mechanisms) did not perform significantly worse compared to the full IIS (i.e. consisting of 1-, 2-, 3- and 4-channel mechanisms for within-fly classification (Fig R3-3). However, the restricted IIS did perform slightly (but significantly) worse for across-fly classification. When taken altogether (both within- and across-fly classification, and with the AIC analysis), we conclude that while 1-channel mechanisms do provide some contribution to the performance of the IIS, they do not drive it. As this result further supports the main claims of the paper, we have decided to provide it in supplementary Text S6 (referred to in Line 648).

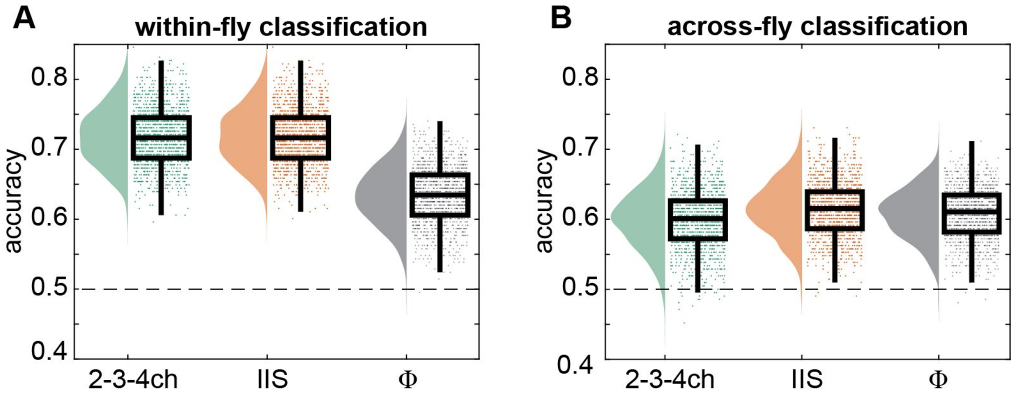

Fig R3-3. Classification accuracy between wakeful vs. anesthetized conditions using the IIS without 1-channel integrated information (green). (A) Within-fly and (B) across-fly classification. We replot the same results for the IIS and system-level integrated information for comparison (the same data as in Fig 5A-B).

> *4) Nature of \Phi measures computed*
>
> *Throughout the paper, the authors refer to the interactions being quantified by their analysis as "causal." This is not true, and it gives a false impression of similarity with the theory in Oizumi 2014. The authors should draw a clear boundary between the theory as presented in Oizumi 2014 (which does very explicitly deal with causal interventions) and the present analysis, which does not. The authors should also clarify (or at least mention) the differences between the measures applied here and IIT 3.0 in the Introduction.*

We agree with you on this point. In addition to the changes we listed in response to the reviewer's summary, we have now also added a discussion section "Differences between perturbation and observation in building the TPM" (Line 678) to draw a clearer line between the theory and our analysis.

> *On a related topic, despite the clear and informative explanation of the computation of \phi, the computation of \Phi is not at all clear in either the text, the figure, or the caption. What does it mean to "best approximate the MICS"? Is this the exact formula given in Oizumi 2014, or some ad-hoc approximation? Please clarify.*

Note that, in our current revision, we changed references to the MICS to CES (cause-effect structure; MICS is the specific CES after searching for the complex). The formula illustrated in Fig. 1H reflects the exact operationalisation of \Phi given in Oizumi 2014 for a given CES and disconnected CES (we previously wrote in Line 173 of the original manuscript - "Fig 1H explains how IIT arrives at … system-level integrated information \Phi"). To help clarify in the main text how \Phi is obtained, we have made the following modifications:

- Line 245 - ADD:
    - "System-level integrated information is the sum of EMDs between the full CES and the CES of the statistically disconnected system, weighted by the integrated information φ of each mechanism in the full CES (as depicted in the calculation between Fig. 1H and 1G; see Methods)."
- Line 250 - ADD:

    - "(i.e. which generates the smallest weighted EMD between the full CES and the disconnected CES)"
- Line 843 - ADD:
    - "(consequently, the distance is weighted by the smaller \phi out of the full CES and disconnected CES)"

> *## Minor comments (most important first)*
>
> *- In several figures (Fig 4A,C, colour bars in Fig 3) the y-axis has a non-uniform spacing, which creates a potentially misleading perception of the data. Please change.*

As log-scale colour bars are unusual, we have decided to change it to a linear scale. We also decided to change Fig 4 to use linear scale.

> *- There were several analysis details that were in figure captions or in the Methods section, and should be more explicitly stated upfront -- for example, the value of \tau and the fact that all measures are averaged across system states.*

We have added these details to the main text:

- Line 193 - ADD:
    - "; we use \tau = 4 ms; we repeated analyses also at τ = 2 ms and 6 ms, see Text S2"
- Line 274 - ADD:
    - "As system-level integrated information and the IIS are obtained for each possible state of the system, we averaged these across states, weighting by the occurrences of each state."

> *- What is the justification for \tau = 4ms?*

We selected 4 ms for two reasons. First, based on the known physiology of synaptic interactions between neurons (Koch 1998 Biophysics of Computation), tau which is too small will not capture causal interactions that maximize integrated information (Hoel 2013 PNAS, 2016 NoC, Marshall 2018 PLoS Comp). Second, given the limited amount of our time series data, larger tau values reduce the number of transitions that we can use to compute the TPM. We chose 4 ms, which balances these two considerations and is within the range of biologically plausible timescales for synaptic interactions in the fly brains. We have added this to the methods:

- Line 805 - ADD: "We use τ = 4 ms as τ which is too small will not capture causal interactions which maximise integrated information, based on known physiology of synaptic interactions (Koch 1998 Biophysics of Computation), and larger τ reduces the number of transitions that we can use to compute the TPM (but see Text S2 for repeated analyses also at τ = 2 ms and 6 ms)."

To exclude the possibility that our results depend heavily on the specific \tau selected, we have added Supplementary Text S2 (referred to in Lines 194, 676, and 808), where we show

essentially the same results we report in the main text, but with tau values of 2 ms and 6 ms. We briefly mention the issue of timescale (and provide our supplementary result) in the Discussion ("Role of system-level integrated information" - Line 664-676).

- In Fig 1C, why isn't the TPM a 4x4 matrix, with 4 future states?

We apologise for the oversight on our end and for omitting an explanation for this. Indeed, transition probability matrices (TPMs) usually describe the probabilities of transitioning from one system state to another system state in a dynamical system. Given that we show a two-channel system in Fig 1, readers would expect the TPM to be a 4x4 matrix.

The state-by-channel TPM is used in IIT 3.0, which assumes that there are no instantaneous interactions among the channels (i.e. the "conditional independence" assumption). In other words, the state of some channel being '1' or '0' at some time point is not affected by the state of other channels at the same time point. This assumption is reasonable for classical physical systems, but may not hold when not all units' interactions are considered (e.g. when there is common input to the system). As it is infeasible to obtain a full description of all parts and interactions of intact brains, this is a limitation of the current IIT 3.0 operationalisation of integrated information (note however that the issue is dealt with and resolved for a previous version of IIT, in Oizumi 2016 PLOS Comp Bio and Oizumi 2016 PNAS, by explicitly incorporating conditional dependence among system parts).

In terms of Fig 1, this conditional independence is expressed by the fact that each column gives the probability of each specific channel being '1' at time t+\tau, given that the system was in some specific state at time t (with each row corresponding to a system state). Thus, the number of columns corresponds to the number of channels being considered. To clarify this (and the conditional independence assumption), we have modified the text:

- Line 180 - ADD:
    - "Each entry of the TPM gives the probability of a given channel taking some state in the future, given the current state of all channels in the system (see Methods)"
- Line 802 - MODIFIED:
    - "For each channel in the set, we computed the empirical probability of being "on" at time $t+\tau$ given the state of the system at time t. This gives a 2^n x n matrix (i.e. a "state-by-channel" matrix)"
- Line 811 - ADD:
    - "The state-by-channel TPM is used in IIT 3.0, which assumes that there are no instantaneous interactions among the channels (i.e. the "conditional independence" assumption). In other words, the state of some channel being '1' or '0' at some time point is not affected by the state of other channels at the same time point. This assumption is reasonable for classical physical systems, but may not hold when not all units' interactions are considered (e.g. when there is common input to the system). As it is infeasible to obtain a full description of all parts and interactions of intact brains, this is a limitation of the current IIT 3.0 operationalisation of integrated information (note however that the issue is dealt with and resolved for a previous version of IIT, in Oizumi 2016 PLOS Comp Bio and Oizumi 2016 PNAS, by explicitly incorporating conditional dependence among system parts)."

- The description of the classifiers could be more clear, for example, by including the number of features and data points used in each classifier. In particular: are all the classifiers except the one based on IIS using just a single predictor?

You are correct here, all classifiers except the one using the whole IIS are using just a single predictor, which we previously mentioned at Line 300 and 547 of the old manuscript. To clarify this, we made the following modifications:

- Line 853 - we now specifically provide the number of features used for the classifiers:
    - "...compare the multivariate IIS **(15-features)** with single mechanism integrated information **(1-feature)** and system-level integrated information **(1-feature)** values"
- Line 879 - ADD:
    - "For accuracy of mechanisms with a given size, we report averaged accuracies across all mechanisms with the given size (e.g. we report 1-channel mechanism accuracy as the average accuracy across all 1-channel mechanisms)."

- I find it unusual that, among mechanisms of size 2, 3 or 4, the wake/anest \phi ratio is reduced with larger mechanism size, but the classification accuracy is increased (while I would intuitively expect these to be positively correlated). Do the authors have any explanation for this?

While the ratio does reduce for larger mechanisms, the variance also decreases, for both the ratio and integrated information values themselves (as can be seen in Fig 4C and D). Specifically, the standard deviations corresponding to Fig 4D (ratio of wake to anesthesia) for 2-, 3-, and 4-channels are 0.0021, 0.0013, and 0.0011, respectively. We now mention this in the manuscript:

- Line 466 - ADD:
    - "..., indicating that the reduction in 4-channel mechanisms, while smaller than that for 2- and 3-channel mechanisms, is more reliable"

- In general, the overall style of figures could be improved (i.e. adjust number of significant figures, uppercase letters in labels, font size, etc).

We have reduced the number of significant digits shown in most of the figures. We decided to keep the significant digits in Fig 1 so that people are able to walk through and verify the calculation of \Phi and \phi themselves if they wish. In general, we have tried to ensure that we follow figure guidelines provided by the journal.

- The format of the references (i.e. title caps vs sentence caps) should be consistent.

Thank you for pointing this out. We will ensure that we follow the guidelines provided by the journal upon acceptance.

- There is a '??' symbol in the caption of Fig. 1 (L.796).

Thanks for pointing this out. This is supposed to be \tau. We have ensured that the character encoding was not lost (for whatever reason) for the revised version.
