# Supplementary material for: Integrated information structure collapses with anesthetic loss of conscious arousal in Drosophila melanogaster
Source: PLoS Comput Biol. 2021 Feb 26;17(2):e1008722. doi: 10.1371/journal.pcbi.1008722 (PMC7946294; doi:10.1371/journal.pcbi.1008722)
Supplement: Attachment — Submitted filename: Response to editor and reviewers.pdf [file pcbi.1008722.s010.pdf]

Dear Prof Daniele Marinazzo,

We thank you for your evaluation of our revised manuscript. We have considered the new comments made by the reviewers, addressing them in the attached reply.

To address your remaining concern on the issue of binarization, we have clarified the difference between our approach and the approach taken by (Hudson PNAS 2014) in our response to Reviewer 2. While the question of how to define states from neural recordings is interesting and important, we think this is too big a question to address in our revision. We have thus expanded a paragraph on this question to the discussion.

With this revision, our manuscript now addresses all concerns raised by the reviewers as you see in our attached responses. We believe our revised manuscript is now strong and clear and is suitable for publication in PLoS Computational Biology.

Regards,

Angus Leung & Naotsugu Tsuchiya

Reviewer #1:

The authors have satisfactorily addressed all of my points.

We would like to thank you for taking the time to go through our manuscript, and for your comments which have helped us improve the manuscript.

Reviewer #2:

This draft of this paper is much improved over the previous draft. In particular, I appreciate the authors' far more nuanced discussion of what their results imply for our understanding of the fly brain and for consciousness more broadly. I also appreciate the authors' clearer discussion of how they computed transition probability matrices, and how those were statistically disconnected for all possible system cuts. My concerns about these points have been satisfactorily addressed.

We would like to thank you for your time and helpful comments. We are glad that we have satisfactorily addressed your initial concerns.

I am still, however, concerned with the validity of computing these matrices from binarized time-series, given that all of the results reported in this paper rest on the validity of this approach. Given that this binarization is used to compute transition probability matrices, the authors' approach essentially assumes that a local field potential can only enter two "relevant" states, with an equal probability of being in one state or the other (though, on this point, I appreciate their demonstration that their results are consistent across different binarization thresholds). As I said in my previous review, this is a problematic assumption for a continuous, non-spiking process like a local field potential. A much more rigorous approach to the same problem was taken by Hudson et al, "Recovery of consciousness is mediated by a network of discrete metastable activity states," PNAS (2014), where discrete transition probability matrices were estimated from local field potential recordings using k-means clustering on principal components estimated from the data. Moreover, given the results reported therein (i.e., that a local field potential can spend more time in some states than in others), a simple binarization at the median for both awake and anesthetized signals (which assumes equal time spent in each state) cannot capture the actual state transitions of the system. The authors' simulation results using a nonlinear autoregressive process do alleviate my concerns along these lines somewhat, and as such I strongly recommend including that analysis in the supplement. But, I would still like to see either a more rigorous discretization approach (for e.g. the one taken by Hudson et al, using k-means clustering of principle components estimated from local field potential data), or at least a more detailed discussion of the limitations of the simple binarization used here.

We agree that the question of how to discretise LFP states is indeed an important one. We would however first like to clarify several points regarding our binarization.

Firstly, while our binarization using the median forces each individual channel to spend equal time in each of two states, this is not necessarily true for the states of 4 channels together spending equal time in each of 16 states (i.e. 2^4 = 16 possible states). Indeed, for a given channel set, we do observe that some of the 16 multichannel states can occur more than others. What integrated information tries to measure is how much of the probability for each of these states cannot be explained by (or reduced into) the probabilities of the constituent subparts of the system. IIT does so through the statistical disconnection (i.e. forced independence via noising) of the channels.

Secondly, we were limited to considering channels with 2 possible states due to limitations of packages used for computing integrated information (PyPhi). While this limitation has very recently been addressed (Gomez 2020 Entropy), we note that when considering more states, the TPMs will potentially become very sparse. For example, if each channel can take 3 states, then a set of 4 channels can take 3^4 = 81 states, 5 times more states than the binarized 4 channels (2^4 = 16). Consequently, many states or transitions may seldom be observed in the multichannel data, leading to unreliable estimates of transition probabilities. Also, this added complexity will exponentially increase computational costs of finding the MIP and other operations necessary to compute integrated information.

In summary, while we agree with the reviewer that this issue requires further investigation, we see it outside the scope of the current paper which is already fairly dense.

We thank you for pointing us to the methods employed in Hudson 2014 PNAS. The two main components in their methods regarding operationalizing states are to: 1) compute power on time windows, instead of at each time step (and employing PCA to reduce dimensionality across frequencies and regions), and 2) cluster of states (based on power spectra across regions).

While we think (1) is a potentially interesting way of operationalizing states, it is somewhat removed from the framework of IIT. Specifically, IIT 3.0 (Oizumi 2014 PLoS Comp Bio) is concerned with moment-by-moment states in the time domain (e.g. voltage; which we have binarized using median split), rather than window-by-window states (e.g. power; however, we have also worked on the spectral version of the integrated information, based on IIT 2.0; Cohen 2020 JNsci Methods). While we think understanding how to analyse and interpret frequency domain data using the IIT 3.0 framework is interesting and potentially important, it is a substantial undertaking based on our own experience for IIT 2.0. Therefore, we consider it outside the scope of the current manuscript.

Regarding (2), we considered defining the states of each channel by clustering voltages (rather than clustering on power). However, we found that the distributions of raw voltages were largely normal (as might be expected from the central limit theorem), giving no motivation for using clustering to define states (Fig R2-1).

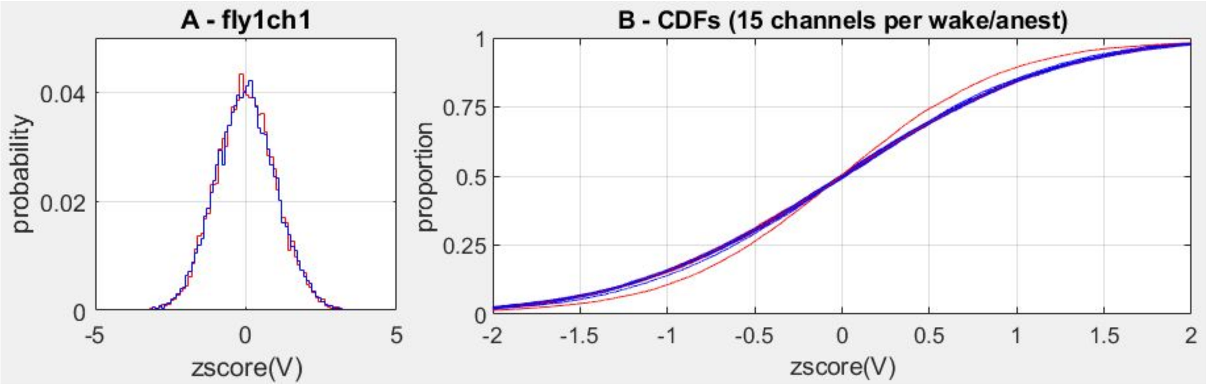

Fig R2-1. Distributions of z-scored voltages from a representative fly. (**A**) Probability normalised histograms of voltages z-scored across 8 epochs of each wake (red) and anesthesia (blue), for the most central channel. (**B**) Cumulative distributions of z-scored voltages for all 15 channels from the same fly, across all 8 trials of each wake and anesthesia (red and blue, respectively; 30 lines in total). Voltages were z-scored per channel and condition. The distributions indicate that none of the channels exhibit specific voltage ranges which can be treated as distinct states.

We have thus decided to expand our discussion on the limitations of our method, specifically regarding the defining of states. As you recommend it, we also provide the nonlinear autoregressive simulation result in supplementary Text S7 (Line 588).

- Line 606 - ADD/MODIFY (relevant modifications **bolded**):
    - “We acknowledge, however, **potential limitations** underlying our recordings **and analyses**. **Firstly**, it is conceivable that, due to the complexity of numerous brain structures in the centre of the brain compared to the relative simplicity of fewer structures in the periphery, signals from a mix of many different structures may have cancelled each other at the raw LFP level. Nonetheless, these central structures may have been more sensitive to the effects of anesthesia. Indeed, we found the effects of anesthesia on system-level integrated information and the IIS to be slightly more reliable for central channel sets (Fig 5C,E). **Secondly, our method of discretizing LFP voltages into binary states may not accurately represent the true space of real states of each of the channels, and also assumes equal probabilities of each state. Further, while IIT 3.0 focuses on moment-by-moment states, other methods, such as considering spectral power in time windows [Hudson 2014 PNAS] may be more useful in describing the states of channels, and so expanding IIT’s framework to consider frequency domain data potentially is a promising avenue for future research [Cohen 2020 JNeuroMethods].** Thirdly, we note that spurious high-order correlations can be found in partially observed multivariate systems and Markovian approximations of non-Markovian systems. These three limitations can be addressed through further investigation, especially with recordings at higher spatial resolutions than LFP, such as optical imaging or neuropixel probes, and expanding of IIT’s theoretical framework.”

Reviewer #3:

I would like to congratulate the authors on a clear improvement of the manuscript. Related to my previous review, statistical reporting has substantially improved (Tables 1 and 2 are especially valuable), and the extra tests and model metrics provide much more information for readers. I am satisfied with the updated discussion of statistical causality, the role of the TPM and its relation with Oizumi2014.

I have recommended the paper for acceptance, although I very strongly suggest the authors consider two further points which have not been addressed so far:

We thank you again for your time and comments which have helped us improve our manuscript. We are glad that we have satisfactorily addressed your initial concerns.

- Most importantly, on the topic of 1-channel mechanisms: the authors should mention (and possibly explore further in future work) the relation between 1-channel \phi and single-channel auto-correlation. Could a change in auto-correlation between conditions explain (some of) the observed results?

We directly compared differences (wake minus anesthesia) in 1-channel φ and difference in single-channel autocorrelation (Fig R3-1). To compute autocorrelation for a given channel, we correlated each LFP time series (of 2.25 s) with itself, shifted by \tau = 4 ms (corresponding to \tau = 4 ms for our integrated information results). Fig R3-1A plots autocorrelation values against 1-channel φ values for one fly during wakefulness. Note that each channel only has one autocorrelation value but multiple 1-channel φ values (each from a different set of 4 channels; 14 choose 3 = 364 channel sets containing the channel; error bars in Fig R3-1A are standard deviations across 364 1-channel φ values). As you can see, some fixed autocorrelation value (x-axis of Fig R3-1A) of a given channel corresponds to multiple, highly varied 1-channel φ values (y-axis). This is expected theoretically, because 1-channel φ has to reflect on how the channel is embedded in and interacts with the other three channels.

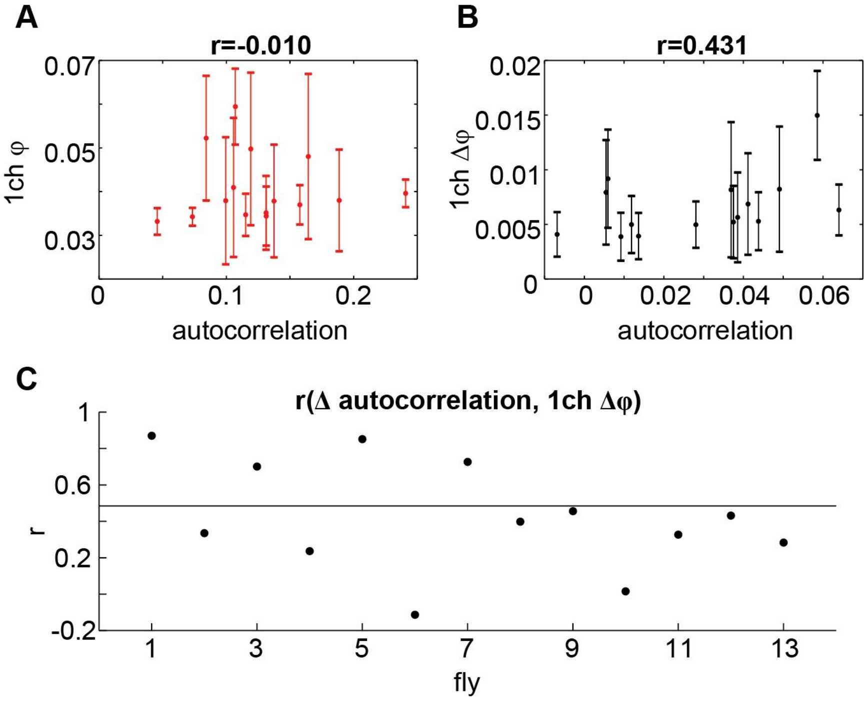

Fig R3-1. Relationship between 1-channel integrated information and autocorrelation, at \tau = 4 ms. (**A**) Single channel autocorrelation plotted against 1-channel integrated information, for a representative fly during wakefulness. Each point corresponds to 1-channel. Error bars are standard deviations of 1-channel φ for a given channel (each channel is contained in 364 out of all 1365 sets of 4 channels). Title gives the correlation coefficient between autocorrelation and 1-channel φ for the fly. (**B**) Difference (wake - anesthesia) in Fisher *z* transformed single-channel autocorrelation (Δ autocorrelation) plotted against difference in 1-channel integrated information (Δφ), for the same fly. Title gives the correlation coefficient between Δ autocorrelation and Δφ for the fly. (**C**) Correlation coefficients between Δ autocorrelation and Δφ for each individual fly. Solid line indicates the average correlation coefficient across flies (coefficients were averaged after Fisher *z* transform, plotted is inverse transform of the mean).

We next subtracted Fisher *z* transformed autocorrelation values during anesthesia from those during wakefulness (Δ autocorrelation). Fig R3-1B shows Δ autocorrelation plotted against Δφ (wake φ minus anesthetised φ), for the same fly as Fig R3-1A. Correlations at each fly, between Δ autocorrelation and average Δφ values of each channel, indicated that there is some positive correlation between the two measures at the group level (Fig R3-1C). We confirmed this using a one-sample t-test comparing Fisher *z* transformed correlation coefficients to 0 ($M = 0.424$, $SD = 0.443$, $t(12) = 4.308$, $p = .001$). In summary, while there seems to be some relationship between the two measures, it is clearly not 1-to-1. As this may be an important characteristic of 1-channel integrated information, and by extension the integrated information structure, we have decided to include these results as new supplementary Text S8, referred to in the discussion (Line 655).

- Line 655 - ADD

    - “While further investigation is necessary to understand our finding regarding 1-channel integrated information **(e.g. such as 1-channel integrated information being potentially related to autocorrelation; see Text S8)** our main results regarding the IIS are unaffected, as we verified that 1-channel mechanisms were not driving its classification performance (Text S6).”

- I find the claims about feedforward systems rather overstated: it is possible to have spurious high-order correlations in multivariate systems when they are partially observed, or when a non-Markovian system is approximated through a Markovian assumption (as is the case here). In this sense, the simulation the authors provided as a reply to Reviewer #2 does not really address the reviewer's concerns, since it is not a non-linear, non-Markovian, or partially observed system.

We agree that partially observed multivariate systems and Markovian approximation of non-Markovian systems can appear to have high-order correlations. We would like to clarify that our simulation is non-linear (specifically, a threshold which must be met before a node can influence another node), which is the original point that we tried to address in this simulation. We are planning future work using simulations to assess how integrated information behaves with the other two conditions (partially observed systems and Markovian approximation of non-Markovian systems), and for now will include these important considerations as limitations in the discussion along with the nonlinear autoregressive simulation results (as Reviewer 2 recommended) in the new supplementary material Text S7.

- Line 588 - ADD
    - “...as system-level integrated information by design should be greater for those areas which have stronger recurrent connectivity as a whole (see Text S7)”
- Line 606 - ADD/MODIFY (relevant modifications **bolded**):
    - “We acknowledge, however, **potential limitations** underlying our recordings **and analyses**. **Firstly**, it is conceivable that, due to the complexity of numerous brain structures in the centre of the brain compared to the relative simplicity of fewer structures in the periphery, signals from a mix of many different structures may have cancelled each other at the raw LFP level. Nonetheless, these central structures may have been more sensitive to the effects of anesthesia. Indeed, we found the effects of anesthesia on system-level integrated information and the IIS to be slightly more reliable for central channel sets (Fig 5C,E). Secondly, our method of discretizing LFP voltages into binary states may not accurately represent the true space of real states of each of the channels, and also assumes equal probabilities of each state. Further, while IIT 3.0 focuses on moment-by-moment states, other methods, such as considering spectral power in time windows [Hudson 2014 PNAS] may be more useful in describing the states of channels, and so expanding IIT’s framework to consider frequency domain data potentially is a promising avenue for future research [Cohen 2020 JNeuroMethods]. **Thirdly, we note that spurious high-order correlations can be found in partially observed multivariate systems and Markovian approximations of non-Markovian systems. These three limitations can be addressed through further investigation, especially with recordings at higher**

spatial resolutions than LFP, such as optical imaging or neuropixel probes, and expanding of IIT's theoretical framework."

As a minor comment, the argument that the authors didn't consider \tau > 6ms because of limited data is rather poor -- increasing \tau by 1ms only reduces the amount of data by 1 sample (judging by the 1kHz sampling frequency), which is not a huge reduction. The authors are free to keep this argument if they like, but I suspect informed readers will find it unpersuasive.

You are correct here. We initially used downsampling as a way of varying \tau, which significantly reduces the number of samples (i.e. halving the sampling rate halves the number of samples). However, in the end, we used the time-sample-skipping scheme. The latter method does not reduce the number of available samples much. We have updated the text to remove this reasoning, and to explicitly mention computational cost as the limiting factor.

- Line 817 - REPLACE:
  - Old: ", and larger τ reduces the number of transitions that we can use to compute the TPM (but see Text S2 for repeated analyses also at τ = 2 ms and 6 ms)"
  - **NEW**: ". A comprehensive search across τ values is infeasible due to computational cost (but see Text S2 for repeated analyses also at τ = 2 ms and 6 ms)"
- Text S2 Para 1 - REPLACE:
  - Old: "Also, given the limited amount of our time series data, larger τ values reduce the number of transitions we can observe in order to build the TPM. Thus we chose 4 ms as our timescale."
  - **NEW**: "Thus we chose 4 ms as our timescale. A comprehensive search across τ values is infeasible due to the computational cost of system-level integrated information."
